# Bridging Endovascular Therapy and Subsequent Surgical Repair for the Treatment of Infected Aneurysms after Percutaneous Cardiac Intervention

**DOI:** 10.1155/2014/814275

**Published:** 2014-07-14

**Authors:** John C. Papakostas, Evgenia Pappa, George N. Kouvelos, Nektario Papa, Miltiadis I. Matsagkas

**Affiliations:** ^1^Department of Surgery-Vascular Surgery Unit, Medical School, University of Ioannina, Ioannina University Campus, S. Niarchos Avenue, 45110 Ioannina, Greece; ^2^Department of Cardiology, General Hospital of Ioannina “G. Hatzikosta,” Makrygianni Avenue, 45445 Ioannina, Greece

## Abstract

Bridging endovascular therapy, accompanied by a second stage open surgical repair was used to treat a rare case of infected aneurysms alongside external iliac artery after a percutaneous cardiac intervention. Because these aneurysms require early treatment, we suggest this approach, in order to avoid immediate, major surgery in a recently symptomatic cardiac and bacteremic patient receiving dual antiplatelet therapy. The approach seems to be safe and durable.

## 1. Introduction

Development of infected arterial aneurysms following percutaneous cardiac interventions is an extremely rare complication [1]. Its treatment, however, is quite challenging, as it has to be applied early in a cardiac patient who is usually under dual antiplatelet therapy, and furthermore, it has to deal with both systemic and local infection.

This report presents a case of such aneurysms on the external iliac artery and suggests a treatment approach that consisted of a first stage bridging endovascular therapy accompanied by a definite open surgical repair some months later.

## 2. Case Report

A 65-year-old man underwent coronary angiography for unstable angina. The sheath was left in place and on the next day a percutaneous transluminal angioplasty (PTCA) with drug eluting stent (DES) deployment was undertaken through a new exchanged sheath, which was removed 10 hours later. All procedures were performed under antibiotic therapy (amoxicillin 1 gr × 3). No complications occurred and patient returned to the Cardiac Intensive Care Unit under dual antiplatelets (aspirin 375 mg and clopidogrel 75 mg).

**Figure 1:**
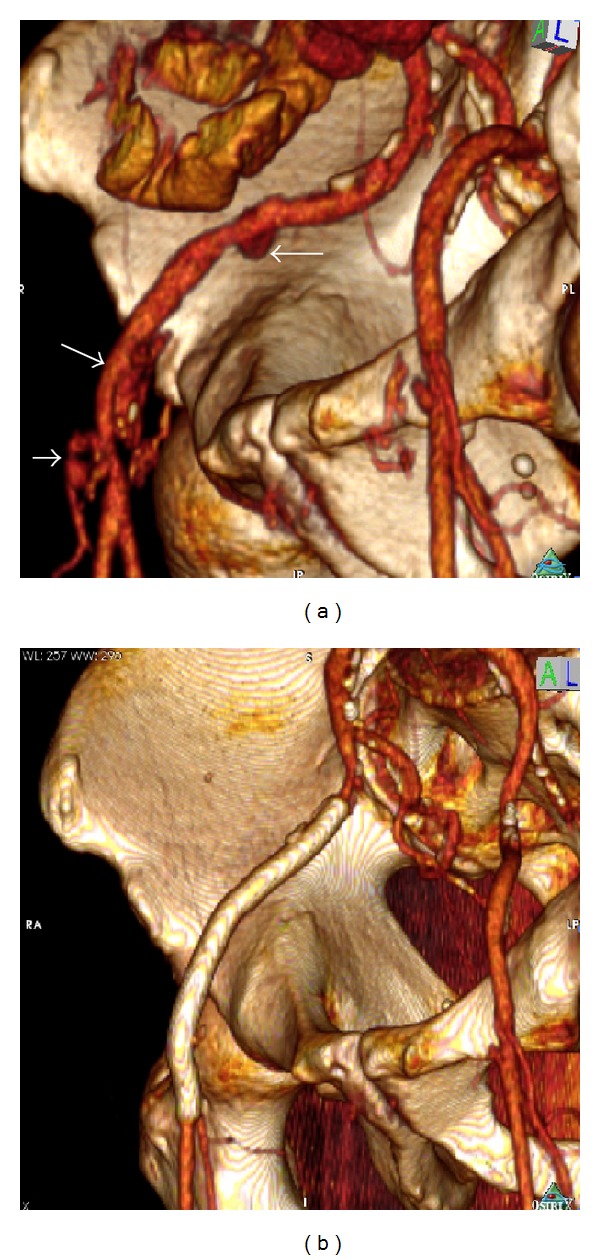
(a) Preoperative computed tomography angiography (CTA) reconstruction, showing the 3 saccular aneurysms of the right external iliac artery (arrows); (b) CTA reconstruction at 3-month followup. In total, two covered stents were deployed with successful exclusion of the infected aneurysms.

The day after the PTCA the patient became septic, and 12 hours later he revealed Osler's nodes in the right leg and Janeway lesions to the right sole. There was purulent drainage from the right groin, without any other organ specific infection signs. Septic endarteritis was highly suspected and the patient was put blindly to antibiotic therapy with vancomycin, rifampicin, and gentamycin, which were later changed to linesolide and rifampicin due to renal function impairment. Groin and blood cultures revealed infection with methicillin resistant staphylococcus aureus which was sensitive to the antibiotics given. Image studies, namely, computed tomography (CT) of abdomen and thorax, computed tomographic angiography (CTA), and colour duplex, were normal. Although septic symptoms were attenuated, fever remained as a single evening wave for the next 3 weeks. At that time point a new CTA was performed, which revealed three small saccular aneurysms of the right external iliac artery (12 mm, 18 mm, and 17 mm in diameter, resp., Figure 1). Ciprofloxacin and ceftriaxone were added to antibiotic therapy. After three days, the patient became afebrile with negative blood cultures and referred to our vascular unit for further treatment.

**Figure 2:**
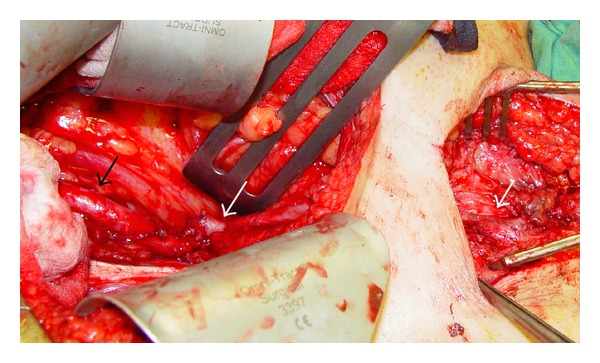
Intraoperative photograph showing the vein bypass (white arrows) from the common iliac bifurcation (black arrow) to the common femoral artery bifurcation.

Under the same antibiotics and dual antiplatelet therapy, we excluded endovascularly the infected aneurysms by deploying two covered stents (Viabahn 7 mm in diameter, 50 and 100 mm in length, Gore, USA) from the orifice of the external iliac artery to the common femoral artery, through percutaneous catheterization of the contralateral (left) femoral artery. The postoperative period was uneventful. The patient remained afebrile with normal blood tests and negative blood cultures and was discharged with antibiotic therapy of Linesolide per os. CTA follow-up at 3 months did not show any problems regarding the stents (Figure 1). Five months later (six after the PTCA) while blood cultures were still negative, we stopped clopidogrel for 7 days and proceeded to open surgery under aspirin 100 mg. Under a right retroperitoneal flank approach we performed complete excision of the right external iliac artery with extensive debridement of the surrounding tissues. A common iliac to common femoral bypass was then applied using greater saphenous vein as conduit (Figure 2). Postprocedural period was uneventful, with palpable pulses at foot arteries and the patient was discharged with dual antiplatelets and antibiotic therapy. Antibiotics were continued for 8 weeks further on.

Two years after the procedure, the patient remains in excellent condition without any signs of infection and with a well working bypass, as confirmed by clinical examination and color duplex ultrasound imaging.

## 3. Discussion

As infected aneurysms are prone to rupture or develop thrombosis with devastating subsequences, early appropriate treatment should be considered mandatory. Traditionally, open surgical repair is the suggested treatment of an infected aneurysm. It offers the opportunity of excision of the infected arterial portion and extensive debridement with subsequent placement of an extra-anatomic bypass for distal perfusion. However, perioperative morbidity and mortality after such operations remain considerable [2]. On the other hand, endovascular treatment gives the option to treat the aneurysm by minimizing periprocedural complications. Although there have been reports of endovascular repair as the only and definite treatment for infected aneurysms, it is not widely suggested [3]. The infected arterial tissue remains in place and in touch with the endograft, and may predispose to the relapse of the infection. Applying endovascular therapy as a bridging approach to a secondary open repair may be a wiser choice and has been proposed for the treatment of infected arterial aneurysms on medically compromised patients or when acute complications are present, such as the presence of secondary aortoenteric fistulas or rupture [4].

Regarding this case, by the time of admission, the patient had a recently reperfused myocardium and was under dual antiplatelet therapy for the DES. These factors made him a high risk candidate for open surgical repair of the infected retroperitoneal area. In order to treat early the rapidly expanding aneurysms and to avoid the periprocedural risks of open surgery we preferred to exclude endovascularly the infected aneurysms and thus continue the antibiotic therapy for a long period without the risk of rupture. In that way we had the opportunity six months after the PTCA to proceed to a definite open surgical repair, discontinuing one of the antiplatelet drugs with relatively low cardiac risk [5], while the patient was free of infection for a long time. With this treatment strategy we utilized the early advantages of endovascular therapy in terms of periprocedural cardiac risks and finally offered the patient a lower risk definite open surgical repair in a second stage.

In our opinion first stage bridging endovascular treatment with a second stage definite open surgical repair may represent a safe and durable approach for the treatment of infected arterial aneurysms in a recently symptomatic cardiac and bacteremic patient under dual antiplatelets.
